# Effectiveness of Acupuncture Used for the Management of Postpartum Depression: A Systematic Review and Meta-Analysis

**DOI:** 10.1155/2019/6597503

**Published:** 2019-03-20

**Authors:** Wei Li, Ping Yin, Lixing Lao, Shifen Xu

**Affiliations:** ^1^Shanghai Municipal Hospital of Traditional Chinese Medicine, Shanghai University of Traditional Chinese Medicine, Shanghai 200071, China; ^2^Guangzhou University of Chinese Medicine, Guangzhou 510006, China; ^3^School of Chinese Medicine, The University of Hong Kong, Hong Kong; ^4^University of Maryland School of Medicine, Baltimore, MD 21201, USA

## Abstract

**Background:**

Previous studies have demonstrated that acupuncture was an effective alternative for treating major depressive disorders. However, the use of acupuncture for the treatment of postpartum depression remains controversial. This review summarizes the most significant studies in the area of acupuncture treatment for postpartum depression and provides a detailed overview of the efficacy of acupuncture for the treatment of postpartum depression.

**Methods:**

We undertook a systematic review of publicly available electronic databases to identify studies that evaluated acupuncture for the treatment of postpartum depression. Our meta-analysis selected randomized controlled trials (RCTs) and quasi-RCTs that reported on the treatment effect of acupuncture on postpartum depression.

**Results:**

Eight prospective trials reporting data on postpartum depression were included in our meta-analysis. The results demonstrated that acupuncture treatment could significantly reduce HAMD scores (SMD: -1.08; 95%CI: -2.11 to -0.05; P=0.040). However, with regard to EPDS, clinical response, and serum estradiol levels, pooled analysis suggested no beneficial effects of acupuncture for postpartum women in EPDS (RR: 1.23; 95%CI: 0.90 to 1.67; P=0.195); clinical response (RR: 1.00; 95%CI: 0.89 to 1.12; P=0.969); and the levels of serum estradiol (SMD: 1.96; 95%CI: -0.01 to 3.93; P=0.051).

**Limitations:**

First, there was relatively high heterogeneity among the studies, except for clinical response. In order to identify the sources of heterogeneity, we divided the studies into subgroups by way of controls. However, heterogeneity still existed, which suggested that it arose from participants rather than controls. Second, the sample size of the studies was small, causing the power of summary results to be low. This may result in over- or underestimating the interpretation of the results. Third, our analysis used pooled data, which restricted us from performing a more detailed analysis.

**Conclusions:**

Our meta-analysis suggested that acupuncture treatment may reduce HAMD scores, while no significant effects on EPDS, clinical response, and serum estradiol levels were observed.

## 1. Introduction

Postpartum depression is a type of mood disorder associated with childbirth, and onset usually begins between day one and four months after delivery [1]. The clinical manifestations of postpartum depression include low energy, extreme sadness, irritability, and suicidal tendencies. Postpartum depression can also lead to subtle negative effects on the newborn child, which may result in a higher incidence of violence during adolescence or adulthood [2].

Given the high prevalence and potentially negative consequences of postpartum depression, it is critical to develop effective treatment strategies for patients with postpartum depression. Currently, the standard therapeutic method for depression is pharmacotherapy. However, most postpartum women are reluctant to take antidepressants due to their potential side effects [3]. Additionally, women with postpartum depression are reluctant to receive psychological and behavioral therapies [4]. Alternative treatment strategies for postpartum depression are gaining prominence, especially acupuncture, and has gained acceptance due to its efficacy and safety.

Previous meta-analysis has demonstrated that acupuncture is a safe and effective treatment for depressive disorders [5, 6]. Furthermore, it is well tolerated and has demonstrated potential anti-depression efficacy in pregnant women [7]. Though several studies have investigated the efficacy of acupuncture on postpartum depression, several of these studies showed inconsistencies in their efficacy. There is a need to determine the efficacy of acupuncture particularly in women with postpartum depression. In this context, we conducted a systematic review and meta-analysis on published studies to evaluate the efficacy of acupuncture treatment for postpartum depression.

## 2. Materials and Methods

### 2.1. Sources

This review and meta-analysis was conducted according to the guidelines of the Preferred Reporting Items for Systematic Reviews and Meta-Analysis Statement (Checklist S1) [8]. We searched PubMed, EmBase, Cochrane Library, ClinicalTrials.gov, China National Knowledge Infrastructure, and Wanfang electronic databases for articles published through September 1st, 2018.

### 2.2. Study Selection

Randomized controlled trials (RCTs) or quasi-RCTs that investigated the effect of acupuncture on postpartum depression women were selected for this study. No limitations were placed on language or publication status (published, in press, or in progress). Keywords and phrases used for queries included “puerperal depression”, “postnatal depression”, “postpartum depression”, “puerperium depression”, “acupuncture”, and “electroacupuncture”. Additionally, we conducted manual searches of the reference lists from all relevant original and review articles to identify additional qualified studies. The study topic, study design, participant's status, intervention, controls, and outcome variables of these studies were used to identify potentially relevant studies.

The literature search was independently handled by two of the authors using the standardized approach. Any inconsistencies during the literature search were scrutinized against the following inclusion criteria: (1) randomized control or quasi-randomized control design was essential; (2) all women were diagnosed with postpartum depression; (3) patients received either acupuncture or traditional therapies; (4) available data concerns the effective rate of acupuncture treatment for extraction; (5) the diagnosis criteria of postpartum depression were based on the International Classification of Diseases (ICD), Chinese Classification of Mental Disorders (CCMD), or the Diagnostic and Statistical Manual of Mental Disorders (DSM). Additionally, the major exclusion criteria were as follows: (1) no available data regarding HAMD scores, EPDS, clinical response, and the levels of serum estradiol; (2) non-case-control studies, case reports, letters, reviewed editorial articles; (3) duplicated publications with previous studies.

### 2.3. Data Collection and Quality Assessment

The collected data comprised the methods and outcome results, the diagnostic instruments and efficacy assessments, treatment regime (monotherapy or additional therapy), controlled conditions (antidepressants and sham acupuncture), and treatment duration. Treatment outcomes were categorized as dichotomous or continuous data. Any adverse effects were noted if they were reported during the meta-analysis. Dichotomous data were mainly response rates, which were generally defined as an improvement in scores on depressive scales or clinical symptoms from baseline to endpoint for acupuncture and controlled groups. Continuous data were baseline-to-endpoint changes in scores on depressive scales that were derived from the Hamilton Rating Scale for Depression (HAMD), Edinburgh Postnatal Depression Scale (EPDS), and serum estradiol levels. Judgments on the methodological qualities of included trials using the Cochrane criteria guidelines [9] were based on the following subscales: concealment of randomization, patients blinding, healthcare providers blinded, data collectors blinded, outcome assessors blinded, and the use of intention-to-treat analysis (Table 2). The data extraction and quality assessments were conducted independently by two of the authors. Any disagreements in assessing the studies were further scrutinized using the above criteria until a consensus was reached.

### 2.4. Statistical Analysis

Dichotomous and continuous data were analysed with risk ratio (RR) and standard mean difference (SMD) with 95% confidence intervals (95% CIs) using the random-effects model. The assumptions made were that the true underlying effect varied among the included trials [10, 11]. Heterogeneity between the studies was investigated using Q statistic, and P values < 0.10 were considered as indicative of significant heterogeneity [12, 13]. Sensitivity analysis was conducted by removing each trial from the overall analysis [14], and subgroup analysis was conducted based on the controls. Visual inspections of funnel plots were performed, and the Egger [15] and Begg [16] tests were used for quantitative evaluate publication bias. All reported P values were 2-sided, and P values < 0.05 were considered statistically significant for all included studies. Statistical analyses were performed using STATA software (version 10.0; Stata Corporation, College Station, TX, USA).

## 3. Results

**Figure 1:**
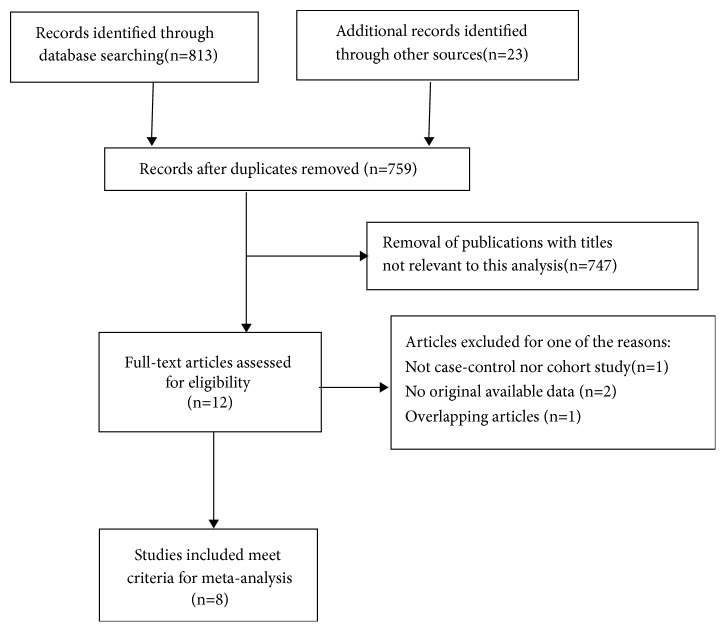
Flow schematic depicting the procedure for retrieving literature and selection of studies.

As shown in Figure 1, a total of 759 potentially useful relevant studies regarding the effect of acupuncture treatment for postpartum depression were initially identified. After screening titles and abstracts of the relevant articles, 747 articles were excluded because they did not meet the inclusion criteria. Afterwards, twelve potentially eligible studies were selected. Upon detailed evaluation, two studies had additional therapeutic benefits for acupuncture on postpartum women [17, 18]. Participants on one of the studies comprised pregnant women and one study did not report primary outcomes [19, 20]. After selection, eight prospective trials were selected for the final meta-analysis [21–28]. A manual search of the reference lists of these studies did not yield any new eligible studies.

In this meta-analysis, a total of 517 participants were selected. All of the patients were diagnosed with postpartum depression according to CCMD-III, HAMD, or DSM-IV criteria, except for two where clinical symptoms were used as the diagnostic criteria [24]. Dou et al. failed to report their diagnosis criteria in their studies [25]. Three of these studies compared acupuncture monotherapy to antidepressants [21, 24, 26], while there were two cohorts that compared acupuncture along with psychological therapy versus antidepressants [23, 25]. There was another study that set electroacupuncture as the intervention with sham acupuncture as the control [27]. Five of these eight selected studies reported clinical response as the outcome [25]. In addition, five studies reported HAMD as the primary parameter used to determine acupuncture treatment efficacy [21–24, 26] (Table 1). The results of the bias within the selected trials are listed in Table 2.

### 3.1. Quantitative Synthesis Results

**Figure 2:**
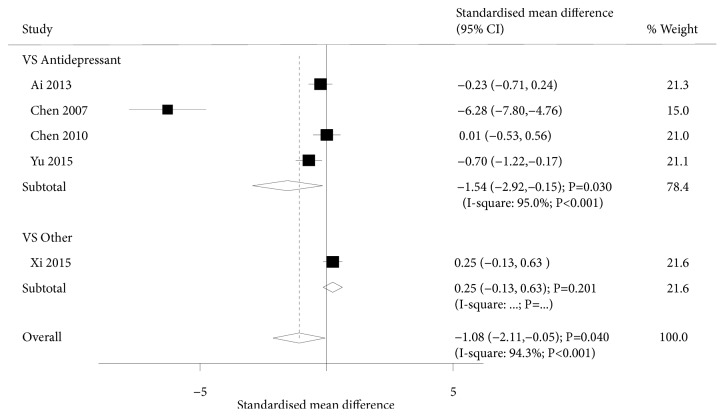
Forest plot displaying the overall effect of acupuncture versus controls in the Hamilton Rating Scale for Depression (HAMD).

A total of 5 trials investigated the effect of acupuncture on HAMD scores in our meta-analysis. The summary results suggested that acupuncture was associated with lower levels of HAMD scores (SMD: -1.08; 95%CI: -2.11 to -0.05; P=0.040; Figure 2) despite significant heterogeneity being observed (P<0.001). Sensitivity analysis suggested that the pooled results were variable due to the small number of included trials. Subgroup analysis suggested that acupuncture significantly reduced the levels of HAMD scores compared to antidepressant drugs (SMD: -1.54; 95%CI: -2.92 to -0.15; P=0.030). However, there were no significant differences between acupuncture and non-drug intervention for HAMD scores (SMD: 0.25; 95%CI: -0.13 to 0.63; P=0.201).

**Figure 3:**
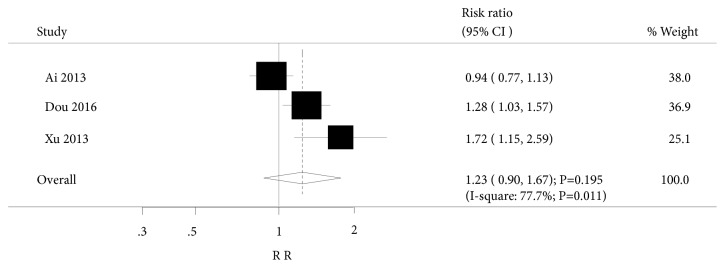
Forest plot displaying the overall effect of acupuncture versus controls in Edinburgh Postnatal Depression Scale (EPDS).

In addition, three trials in our meta-analysis investigated the effect of acupuncture on EPDS, and two trials of these included studies compared acupuncture with antidepressants, while one compared acupuncture with placebo. Pooled analysis suggested no beneficial effects of acupuncture were observed in postpartum women (RR: 1.23; 95%CI: 0.90 to 1.67; P=0.195; Figure 3) and were associated with higher heterogeneity (I^2^=77.7%; P=0.011). Additionally, subgroup analysis suggested that acupuncture was associated with a higher incidence of EPDS when compared to placebo (RR: 1.72; 95%CI: 1.15 to 2.59; P=0.009). This effect was not observed when compared to antidepressant drugs (RR: 1.09; 95%CI: 0.80 to 1.48; P=0.585).

**Figure 4:**
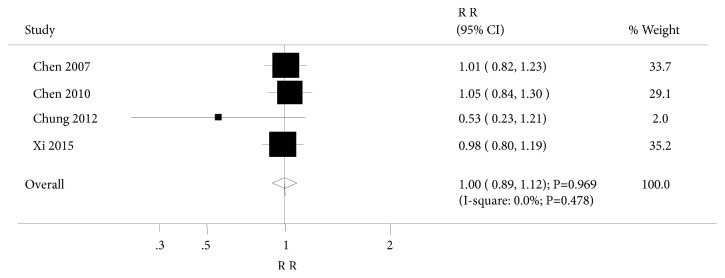
Forest plot displaying the overall effect of acupuncture versus controls for clinical response.

Four trials investigated the effect of acupuncture on clinical response. Summary results suggested no significant differences between acupuncture and controls for clinical response (RR: 1.00; 95%CI: 0.89 to 1.12; P=0.969; Figure 4). There was no evidence of heterogeneity across the studies. After sequential exclusion of every study, none of the studies affected the pooled analysis. Subgroup analysis was based on control types and consistent with the overall analysis results.

**Figure 5:**
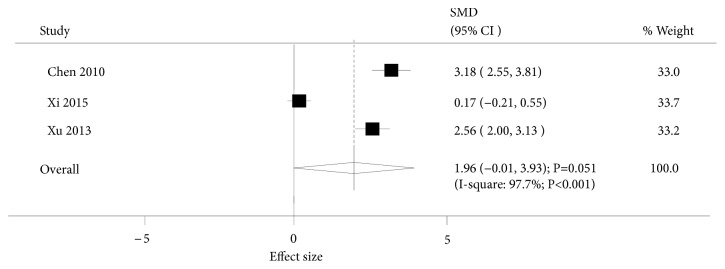
Forest plot displaying the overall effect of acupuncture versus controls for serum estradiol levels.

Three trials investigated the effect of acupuncture on serum estradiol levels. Pooled analysis results indicated that there was no association between acupuncture and the levels of serum estradiol (SMD: 1.96; 95%CI: -0.01 to 3.93; P=0.051; Figure 5). However, subgroup analysis suggested that women who received acupuncture treatment were associated with higher levels of serum estradiol (SMD: 3.18; 95%CI: 2.55 to 3.81; P<0.001) compared to women on antidepressant drugs. Conversely, no differences were observed when compared to women who did not receive drug intervention (SMD: 1.36; 95%CI: -0.99 to 3.70; P=0.257).

**Figure 6:**
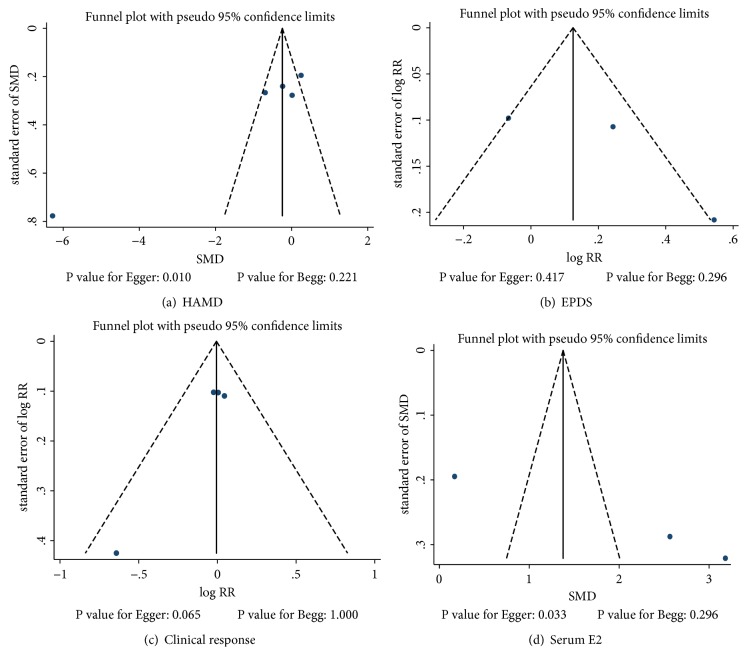
Funnel plots for Hamilton Rating Scale for Depression (HAMD), Edinburgh Postnatal Depression Scale (EPDS), clinical response, and serum estradiol levels.

Review of the funnel plots could not rule out potential publication bias for HAMD scores, EPDS, clinical response, or serum estradiol levels (Figure 6). However, we did note potential evidence of publication bias for HAMD scores using Egger test (P=0.010). There was no evidence of publication bias for EPDS using Egger test. The Egger test showed potential publication bias (P=0.065) and potential evidence of publication bias for serum estradiol levels (P=0.033).

## 4. Discussion

Growing evidence has demonstrated the beneficial effect of acupuncture treatment for postpartum depression. A previous meta-analysis [6] suggested that acupuncture had a significantly greater overall effective rate compared to controls. Moreover, acupuncture significantly increased estradiol levels compared to controls. Regarding HAMD and EPDS scores, no differences were observed between the two groups. However, due to the limited number of participants, such associations remain controversial. Therefore, we aimed to further investigate the association between the effect of acupuncture treatment and postpartum depression in this meta-analysis. Our meta-analysis demonstrated that acupuncture could significantly reduce HAMD scores in postpartum women, but no additional benefits from acupuncture treatment were observed when compared to conventional treatments. The treatment effects of acupuncture on postpartum depression differed when compared to different controls. Subgroup analysis suggested that acupuncture could significantly reduce the levels of HAMD scores, and higher levels of serum estradiol were observed in acupuncture treated versus patients prescribed antidepressant drugs. Furthermore, acupuncture significantly increased the incidence of EPDS as compared to placebo. Unlike the efficacy of acupuncture on other depression diseases such as major depressive disorder and post-stoke depression or even depression related insomnia [29], the treatment efficacy of acupuncture on postpartum women was lower compared to other treatment methods.

More than half the selected studies (5/8) [21, 24, 27] reported on the details of randomization before treatment. Only one study performed allocation concealment, while the majority of the trials did not involve allocation information [27]. This uncertainty may lead to a bias for selection and detection and could have a negative consequence on interpreting the strength of the results. In addition, there was difficulty in utilizing the double-blind method to carry out acupuncture treatment; however, the sham needle acupuncture method could have been a viable alternative. In addition to these complications in interpreting the data, loss of follow-up of the participants happened in two studies.

Our meta-analysis showed that the overall effects of acupuncture were similar to other treatment methods when the data was summarized as absolute mean differences. Continuous data had higher variability than expected. Hence, SMD was used to evaluate the treatment efficacy of acupuncture. Several studies have reported inconsistent results in their summaries, which made the analysis more complicated. Ai et al. reported that there were no significant differences between acupuncture and psychotherapy versus antidepressant drugs for HAMD and EPDS; however, they maintained that combining acupuncture with psychotherapy would be regarded as an effective alternative treatment strategy [23]. Chen et al. suggested there were no significant differences between acupuncture and antidepressant drug treatment for HAMD levels; however they indicated that women who received acupuncture had a lower risk of adverse events [21]. Xi et al. reported no significant differences between acupuncture and psychotherapy in HAMD or serum estradiol levels. However, they suggested that combining acupuncture with psychotherapy would result in higher HADM, lower self-rating depression scale scores, and self-rating anxiety scale scores [22]. Dou et al. reported that women who received acupuncture had significant increase in the incidence of EPDS, but critical data was missing in their report to verify their conclusions [25]. The findings of the study conducted by Xu et al. were consistent with Dou et al. [28]. Although our analysis suggested no relationship between acupuncture and serum estradiol levels, two of the included trials reported higher levels of serum estradiol in women who received acupuncture. Previous studies had reported acupuncture treatment would result in the increase of estradiol levels. However, Xi et al. showed no such differences observed in their study [22], which may be due to the differences in the interventional or control groups.

Though our meta-analysis revealed that acupuncture had a lower efficacy compared to other antidepression treatments, symptoms of depression relief were observed in the majority of the studies analysed. These findings indicate that although the efficacy of acupuncture was lower compared to antidepressants, the benefits of acupuncture were still attractive to women with postpartum depression due to their negligible side-effects.

There were several limitations to our study: first, there was relative high heterogeneity among the studies, except for clinical response. We divided the studies into subgroups by way of controls in order to identify sources of heterogeneity. Heterogeneity still existed, suggesting that the source of heterogeneity could be due to participant selection rather than the controls. Second, the sample size of the studies was small, causing the power of summary results to be lower. This would result in over- or underestimating the interpretation of the results. Third, unpublished studies would likely include increased proportions of negative results, but by definition we were not aware of them and could not include them here. In addition, our analysis used pooled data, which restricted us from performing a more detailed analysis.

## 5. Conclusions

In summary, despite the limitations mentioned above, this current systematic review suggests that acupuncture could reduce HAMD scores. However, EPDS, clinical response, and serum estradiol levels were unaffected by acupuncture treatment. Furthermore, our analysis revealed that treatment efficacy was variable and depended on the control types used. Large-scale and well-designed RCTs are required before a conclusive statement could be made regarding the efficacy of acupuncture for the treatment of postpartum depression.

## Figures and Tables

**Table 1:** Characteristics of the selected studies used in the meta-analysis.

Authors	Sample size	Diagnostic Criteria	Intervention	Controls	Outcomes
Chen 2010 [21]	52	CCMD-III, HAMD	Acupuncture	Antidepressant (Fluoxetine)	Clinical Response, HAMD, Estradiol
Xi 2015 [22]	104	CCMD-III, HAMD	Acupuncture	Psychological therapy	HAMD, Estradiol
Ai 2013 [23]	70	CCMD-III	Acupuncture and psychological therapy	Antidepressant (Citalopram)	Clinical Response, HAMD, EPDS
Yu 2015 [24]	60	Clinical Symptoms	Acupuncture	Antidepressant (Fluoxetine)	HAMD
Dou 2016 [25]	80	Unknown	Acupuncture and psychological therapy	Antidepressant (Fluoxetine)	Clinical Response, EPDS
Chen 2007 [26]	41	DSM-IV	Acupuncture	Antidepressant (Probucil)	Clinical Response, HAMD
Chung 2012 [27]	20	DSM-IV, HDRS17	Electroacupuncture	Sham acupuncture	Clinical Response, EPDS,
Xu 2013 [28]	90	DSM-IV	Acupuncture	Placebo	EPDS, Estradiol

HAMD: Hamilton Rating Scale for Depression; EPDS: Edinburgh Postnatal Depression Scale.

**Table 2:** Assessment for risk of bias in the randomized clinical trials.

Study	Concealment of Randomization	Patients Blind	Healthcare Providers Blinded	Data Collectors Blinded	Outcome Assessors Blinded	Intention-to-treat analysis
Chen 2010 [21]	Yes	No	No	No	No	Yes
Xi 2015 [22]	Yes	No	No	No	No	Yes
Ai 2013 [23]	Yes	No	No	No	No	Yes
Yu 2015 [24]	Yes	No	No	No	No	Yes
Dou 2016 [25]	NA	No	No	No	No	Yes
Chen 2007 [26]	NA	No	No	No	No	No
Chung 2012 [27]	Yes	Yes	No	NA	NA	Yes
Xu 2013 [28]	Yes	No	No	No	No	NA

## Data Availability

The datasets supporting our results are included within this article.
